# Father and Sibling Involvement in Home Rehabilitation: Longitudinal Effects on Infant Development and Maternal Wellbeing in Bulawayo, Zimbabwe

**DOI:** 10.1111/cch.70292

**Published:** 2026-05-06

**Authors:** Precious Madzimbe, Soraya Maart, Jermaine Dambi, Lieselotte Corten

**Affiliations:** ^1^ Department of Health and Rehabilitation Sciences, Faculty of Health Sciences University of Cape Town Cape Town South Africa; ^2^ Department of Physiotherapy and Occupational Therapy United Bulawayo Hospitals Bulawayo Zimbabwe; ^3^ Department of Rehabilitation Sciences, Faculty of Medicine and Health Sciences University of Zimbabwe Harare Zimbabwe; ^4^ School of Education, Sport and Health Sciences University of Brighton Brighton UK

**Keywords:** father involvement, home rehabilitation, longitudinal cohort study, neuro‐developmental delay, siblings, Zimbabwe

## Abstract

**Objective:**

To determine whether extending home‐based rehabilitation beyond the mother‐only model to include fathers and siblings is associated with improved developmental outcomes in infants with neuro‐developmental delay (NDD) and maternal wellbeing in an urban low‐resource setting.

**Methods:**

A prospective observational cohort study was conducted at two public neuro‐developmental clinics in Bulawayo, Zimbabwe. Infants aged 3–6 months (*N* = 481) and their mothers were followed for 3 months across three naturally occurring caregiver participation groups: mother‐only, mother–father and mother–father–sibling. Developmental outcomes were assessed using the Bayley Scales of Infant and Toddler Development, Third Edition (BSID‐III), and maternal wellbeing was measured using Global Quality of Life (QoL) and Mental Health Check‐In Visual Analogue Scales. Group differences were analysed using ANCOVA, repeated‐measures ANCOVA and multiple linear regression adjusting for baseline scores.

**Results:**

Infants in the mother–father–sibling group demonstrated significantly higher baseline‐adjusted BSID‐III cognitive scores at 3 months than the mother‐only group (*p* < 0.05), with the largest effect observed in this group. Changes in language, motor, socioemotional, and adaptive domains followed the same direction but showed smaller and less consistent effects, with limited pairwise significance after adjustment. Maternal mental health improved significantly over time across groups, while QoL showed small but statistically significant gains only on repeated‐measures analysis.

**Conclusion:**

Involving fathers and siblings in home‐based rehabilitation was associated with selective cognitive gains in infants and better maternal mental health over 3 months. These findings provide preliminary support for the integration of father‐ and sibling‐inclusive, family‐centred rehabilitation models for paediatric neurorehabilitation in similar low‐ and middle‐income settings.

## Summary

- In routine neuro‐developmental clinics, actively involving fathers and siblings alongside mothers in home‐based rehabilitation may support selective cognitive gains in infants with neuro‐developmental delay over a three‐month period.
- Maternal mental health improved most where caregiving was shared, suggesting that multi‐caregiver participation can reduce the psychological burden on mothers and strengthen family capacity for sustained home practice.
- Family‐centred care in low‐resource settings should move beyond mother‐only models by incorporating father‐ and sibling‐inclusive guidance within clinic education, follow‐up systems and communication pathways.
- Scalable implementation may require service adaptations such as flexible clinic hours, caregiver‐friendly messaging and role‐specific home programme instructions to enable fathers' participation without compromising household livelihoods.
- Longer follow‐up and objective measures of dose/fidelity of home practice, sibling roles (age and responsibilities) and maternal socioeconomic context are needed to clarify mechanisms and determine whether early cognitive gains translate into broader developmental benefits.

**Abbreviations**
ARSS: at risk surveillance system
BSID‐III: Bayley Scales of Infant and Toddler Development, Third Edition
FCC: family‐centred care
GQL: Global Quality of Life
HADS: Hospital Anxiety and Depression Scale
LMICs: low‐ and middle‐income countries
MANSA: Manchester Short Assessment of Quality of Life
NDD: neuro‐developmental delay
PGD: perceived global distress
QoL: quality of life
VAS: Visual Analogue Scale

## 1 Introduction

Children under five in low‐ and middle‐income countries (LMICs) bear a disproportionate burden of neuro‐developmental disabilities, with nearly 95% of all affected children residing in these regions (Olusanya et al. 2018). Although global prevalence has declined modestly, the burden of neuro‐developmental delays (NDDs) remains high, highlighting the need for scalable strategies embedded within health systems. Family‐centred care (FCC), which positions families as active partners, is associated with improved rehabilitation outcomes and aligns with the Nurturing Care Framework; however, operational FCC models remain limited in African contexts (Britto et al. 2017; Chow et al. 2024; King et al. 2017). In practice, inclusive FCC models involve structured caregiver guidance, shared caregiving roles and participation of multiple family members in home rehabilitation activities and parent training.

In sub‐Saharan Africa, paediatric rehabilitation is largely mother‐focused, shaped by health system priorities and sociocultural norms that position mothers as primary caregivers. This model often excludes fathers and siblings from formal rehabilitation frameworks, limiting caregiving diversity and hindering inclusive, family‐centred practice (Nsamenang 2010; Richter and Morrell 2006). Addressing the lack of structural integration of broader family roles in rehabilitation policy and services could foster shared responsibilities and enhance developmental support in low‐resource settings (Abubakar et al. 2008).

Emerging evidence suggests that fathers' sensitive engagement in caregiving of children with NDD supports socioemotional and cognitive outcomes, while siblings contribute both practical and emotional support that benefit children and families (Cuskelly et al. 2023; Diniz et al. 2021). Systematic reviews further associate father involvement with favourable developmental and behavioural trajectories across childhood (Rollè et al. 2019; Sarkadi et al. 2008). However, longitudinal evidence quantifying the added value of structured father and sibling participation in home rehabilitation remains scarce, particularly in sub‐Saharan Africa where health systems rarely extend support beyond maternal caregivers (Chow et al. 2024; King et al. 2017).

Therefore, this study aimed to investigate whether extending caregiver participation beyond mothers to include fathers and siblings is associated with improved developmental outcomes in infants with NDD and maternal wellbeing. We compared three participation groups (mother‐only, mother and father and mother–father–sibling triads) over a 3‐month period, using BSID‐III domains, maternal quality of life and mental health indices. We hypothesised that broader caregiver participation would be associated with greater developmental gains and improved maternal wellbeing, consistent with evidence that enhanced family engagement increases therapeutic intensity, reduces caregiver stress and supports developmental progress (Jeong et al. 2021; Madzimbe et al. 2024).

## 2 Methods

### 2.1 Design and Setting

This study used a prospective observational cohort design (MacGinty et al. 2020; Springer et al. 2018) and was conducted at United Bulawayo Hospitals (UBH) and Mpilo Central Hospital (MCH), Bulawayo, Zimbabwe. Infants were followed over a 3‐month period, identified as a clinically meaningful duration for detecting milestone progression in children receiving rehabilitation (Dambi and Jelsma 2014; Fan et al. 2021; Harbourne et al. 2018; Ministry of Health and Child Care 2021). Group allocation was observational, reflecting real‐world patterns of home programme participation (mother‐only, mother–father and mother–father–sibling). The home programme comprised daily play‐based exercises prescribed by rehabilitation professionals (physiotherapists, occupational therapists and rehabilitation technicians) and demonstrated during routine weekly clinic sessions at both UBH and MCH.

The frequency of clinic attendance was similar across the two hospitals, as both operate scheduled neuro‐developmental clinics once per week, during which caregivers received review and reinforcement of the home programme. During each clinic visit, the prescribed activities were re‐demonstrated, clarified and progressed where appropriate over the three‐month follow‐up period. Caregivers were encouraged to ask questions, and they were permitted to take notes, photographs or short video recordings of the demonstrations using their personal mobile phones to facilitate recall and fidelity of implementation at home. Because the clinic schedules and frequency of reviews were comparable across both hospitals, exposure to demonstrations was considered similar between groups. The prescribed home programmes were identical across all groups; only the participating caregivers differed.

In groups involving both parents, activities were performed jointly or alternated based on availability. In the mother–father–sibling group, siblings aged 7 years and above participated under parental supervision in simple tasks such as play stimulation, positioning and assisting with toys or reaching activities. Children in this age range generally have sufficient cognitive understanding, attention and motor skills to follow simple instructions and safely assist with supervised home‐based rehabilitation activities. No changes were introduced to participants' usual rehabilitation management; the study simply observed these natural participation patterns over the 3‐month period to compare developmental outcomes across groups while adjusting for baseline values (Mann 2003; Szklo and Nieto 2019).

Caregiver participation patterns were verified through caregiver interviews and confirmation during clinic reviews. Participation classification was based on regular involvement in the home programme (at least four times per week), as reported by caregivers and confirmed by the research assistants during follow‐up visits.

UBH and MCH run weekly neuro‐developmental clinics serving approximately 1000 children annually, with about 20–30 cases per week for each hospital involving conditions such as cerebral palsy, global developmental delay, autism spectrum disorder, and genetic syndromes. When attending these clinics, children are screened using the Health Worker Screen Chart (Ministry of Health and Child Care [MoHCC], 2021) and managed by interdisciplinary teams. This structured, high‐volume setting provides an appropriate platform to investigate father and sibling participation in rehabilitation.

### 2.2 Participants

The study population comprised mothers and their infants with clinically diagnosed NDD (e.g., cerebral palsy, hydrocephalus, spina bifida) attending routine follow‐up at UBH and MCH.

#### 2.2.1 Inclusion Criteria

The following are the inclusion criteria: infants aged 3–6 months at baseline, as this age represents an early neuro‐developmental period when delays can be reliably identified and when home‐based rehabilitation may influence emerging developmental trajectories, with NDD as recorded in hospital clinical records using ICD‐10 diagnostic classifications assigned by paediatricians, including conditions such as cerebral palsy, hydrocephalus and spina bifida, which are commonly associated with developmental impairment; attendance with the primary caregiver; maternal literacy in English, Shona or IsiNdebele.

#### 2.2.2 Exclusion Criteria

Exclusion criteria include the following: severe behavioural or regulatory difficulties precluding reliable assessment (e.g., persistent inconsolable crying, extreme irritability or inability to engage with assessment materials), acute illness (> 38°C fever or respiratory distress) and profound multi‐system impairments precluding valid BSID‐III assessment. Mothers with significant cognitive impairment precluding consent were also excluded.

Disability severity (mild, moderate, severe, profound) was classified according to the attending clinician's functional assessment recorded in hospital clinical notes, reflecting the overall developmental and functional status of the child at enrolment.

### 2.3 Sampling and Sample Size

Group allocation was non‐random and based on naturally occurring family participation patterns, consistent with the observational design. To achieve balanced representation across these participation groups, quota sampling was used, followed by simple random selection of participants within each quota (Nikolopoulou 2022). Quota sampling was selected to ensure adequate representation of the three caregiver participation patterns, which were unevenly distributed in routine clinic attendance.

The sample size was estimated at 150 infants per group (total *N* = 450) using a pairwise comparison of means, assuming a medium effect size (d = 0.4, power = 0.80, α = 0.05). To accommodate attrition, oversampling was applied to reach *N* = 488 (approximately 8% surplus), consistent with cohort study recommendations (Borm et al. 2007; Fewtrell et al. 2008). Final quotas were informed by clinic registers in consultation with a biostatistician, yielding a final analytic sample of *N* = 481 after attrition (98.6% follow‐up). This reflects strong follow‐up compliance.

### 2.4 Data Collection Procedures

Eligible dyads were identified during routine clinic visits. The study was clearly explained to caregivers, and written informed consent was obtained prior to inclusion. All participating families were already enrolled in these home‐based rehabilitation programmes before the commencement of the study. The programmes were standard clinical management plans designed by the departmental rehabilitation professionals and implemented as part of routine care. No new intervention was introduced by the research team. Instead, participants were observed within their naturally existing family participation structures.

During enrolment, the first research assistant conducted a brief history‐taking to determine caregiver participation patterns during routine patient subjective assessment, which were verified by the second research assistant and used to assign participants to one of the following three groups. Caregivers were asked structured questions regarding who regularly assisted with the home programme, how frequently they participated and whether participation occurred consistently during the week. Only caregivers who were reported to participate regularly in home activities were considered for classification in the multi‐caregiver groups.

- Home programme administered mainly by the mother.
- Home programme administered jointly by the mother and father.
- Home programme administered jointly by the mother, father and siblings.

At the baseline assessment, the lead investigator, a paediatric physiotherapist trained in administering the Bayley Scales of Infant and Toddler Development, Third Edition (BSID‐III), performed developmental assessments in a quiet, designated therapy cubicle within the neuro‐developmental clinic to ensure privacy and minimise distractions. The research assistant, who had been adequately trained by the principal investigator, assisted in completing the data‐collection sheets. Each assessment required between 50 and 90 min, depending on the child's age and level of cooperation. Short breaks were permitted during the assessment where it is required to accommodate feeding, soothing or rest periods for the infant.

Assessments were conducted before or immediately after each family's routine physiotherapy review, depending on the infant's state of alertness and feeding schedule. All BSID‐III evaluations followed standardised administration and scoring protocols, and test materials were sanitised between participants. A single assessor, blinded to group classification, conducted all assessments to enhance measurement reliability.

### 2.5 Use of Artificial Intelligence (AI) Tools

AI‐assisted language editing was used in the preparation of this manuscript. Anthropic Claude (large language model) was used solely for language editing and clarity, including grammar, punctuation and phrasing. No AI tool was used to generate research content, study design, data, analyses, results or interpretations. The authors reviewed and take full responsibility for the final content.

### 2.6 Instruments and Measures

#### 2.6.1 Sociodemographic Details

A researcher‐administered sociodemographic questionnaire was used to collect key background information, including the child's and mother's dates of birth, the child's biological sex and the child's medical diagnosis. Additional variables included maternal education, employment status, residential area, medical aid coverage, family history of NDD and financial adequacy as reported by caregivers. Financial adequacy was assessed through caregiver self‐report and categorised as *very inadequate*, *somewhat inadequate*, *moderate*, *somewhat adequate* or *very adequate*, reflecting the caregiver's perceived ability to meet household financial needs. These variables were included to ensure that the study sample was accurately characterised and that sociodemographic variations could be appropriately considered in the interpretation and reporting of results.

#### 2.6.2 Infant Neuro‐Development (Primary Outcome)

The Bayley Scales of Infant and Toddler Development, Third Edition (BSID‐III) assesses developmental functioning in children aged 1 to 42 months across five domains: cognitive, language, motor, socioemotional, and adaptive behaviour. Each subtest begins once the child successfully completes three consecutive items and ends after five consecutive failures. Raw scores are calculated as the total number of items credited and are converted using age‐specific norms into scaled scores (M = 10, SD = 3) and composite scores (M = 100, SD = 15). These composite scores classify performance based on the child's age as very superior (≥ 130), superior (120–129), high average (110–119), average (90–109), low average (80–89), borderline (70–79) or extremely low (≤ 69). The socioemotional and adaptive behaviour domains are completed through caregiver‐reported questionnaires, and their responses are similarly converted into standardised composite scores to provide an overall developmental profile (Bayley 2006).

The BSID‐III has demonstrated strong reliability and validity across diverse settings, including China, Zimbabwe and South Africa, confirming its cross‐cultural applicability in LMICs. In China, Hua et al. (2019) reported inter‐item consistency (Guttman split‐half) above 0.80, and test–retest and inter‐rater ICCs exceeding 0.90, with criterion‐related correlations (vs. the Gesell Developmental Schedules) mainly above 0.80. In South Africa, Rademeyer and Jacklin (2013) found that urban Black African infants' mean composite scores (*M* = 103.4) were significantly higher than the United States' norms (*M* = 100), with subtest means of 99.7 (cognitive), 106.8 (language) and 103.5 (motor). Ballot et al. (2017) affirmed the suitability of the BSID‐III for urban South African cohorts. In Zimbabwe, Hutchings and Potterton (2013) reported measurable NDDs among HIV‐exposed infant cohorts, supporting the feasibility of standardised assessment and emphasising the urgency of early intervention.

#### 2.6.3 Maternal Outcome (Secondary Outcome)

Maternal QoL was measured with the Global Quality of Life (GQL) scale, which has been demonstrated to have satisfactory reliability and construct validity (Ivarsson et al. 2010). Specifically, Ivarsson et al. (2010) reported satisfactory test–retest reliability for the GQL and strong concurrent validity with the Manchester Short Assessment of Quality of Life (MANSA) ‘Life as a Whole’ item (*r* = 0.85), with complementary perceived global distress (PGD) evidence showing MANSA convergence (*ρ* = 0.59) and test–retest reliability (*ρ* = 0.75) (Ivarsson et al. 2011).

Maternal mental health was assessed with the 1–10 Check‐in Visual Analogue Scale (VAS), validated as a reliable stress/mental‐health measure in clinical and community samples, showing acceptable agreement with the Perceived Stress Scale and good discrimination [sensitivity (*Se*) = 0.74; specificity = 0.93] (Lesage and Berjot 2011) and construct validity via correlations with Hospital Anxiety and Depression Scale (HADS) anxiety/total/depression of 0.66/0.65/0.45 (Lesage et al. 2012). Both measures have shown feasibility in LMICs for caregiver‐reported wellbeing; the Adult Carer Quality of Life Questionnaire adapted for Malaysia reported composite reliability (*CR*) values of 0.77–0.91 and test–retest *ICC* = 0.86 (Khan et al. 2025).

#### 2.6.4 Statistical Analysis

Descriptive statistics summarised baseline characteristics, with continuous variables reported as means and standard deviations or medians and interquartile ranges depending on distribution, and categorical data as frequencies and percentages. Baseline group comparisons were performed using one‐way ANOVA for continuous variables and χ^2^ tests for categorical variables. Data normality was assessed using *Shapiro–Wilk* tests, histograms, and Q–Q plots; given the large sample size per group (> 150), the Central Limit Theorem supported the use of parametric analyses.

For continuous outcomes (BSID‐III, quality of life and mental health), ANCOVA and repeated‐measures ANCOVA models were used, with baseline values entered as covariates. Outcome scores at follow‐up served as the dependent variables, while group, time and the time × group interaction term were modelled as independent effects to assess between‐group differences in change over time. With *N* = 481 and four parameters estimated (intercept, two group indicators and the baseline covariate), the residual degrees of freedom were 481–4 = 477 for BSID‐III models; therefore, omnibus tests of the group effect are reported as *F*(2, 477). Omnibus ANCOVA *F*‐tests were first conducted to determine whether any overall group differences existed across domains, followed by pairwise comparisons of estimated marginal means (EMMs) using the Tukey post hoc adjustment for multiple testing.

Linear mixed‐effects models with random intercepts were fitted using maximum‐likelihood estimation as sensitivity analyses to validate the primary findings and to account for within‐subject correlations over time; these analyses yielded results consistent with the main ANCOVA‐based models. Only participants with complete data at both baseline and follow‐up assessments were included in the final analysis, resulting in a complete‐case dataset with no missing values after attrition (*N* = 481). Analyses were performed in Stata Statistical Software, Release 17 (StataCorp 2021).

## 3 Results

### 3.1 Sociodemographic Characteristics of Mothers and Children by Group

Table 1 summarises the sociodemographic and clinical characteristics of mothers and children across the three participation groups. All infants (*N* = 481) completed the BSID‐III assessment and all mothers (*N* = 481) completed the Global QoL and Mental Health Check‐in at baseline and after 3 months. The number of children with NDD per group was as follows: Group 1–172 (35.8%), Group 2–154 (32.0%) and Group 3–155 (32.2%). Groups were broadly comparable at baseline; one‐way ANOVA (continuous) and *χ* ^*2*^ tests (categorical) showed no significant differences (*p* > 0.05) except for maternal employment status (*p* = 0.003).

**TABLE 1:** Sociodemographic characteristics of mothers and children by group, *N* = 481.

Variable	Group 1 *N* (%) = 172 (35.8)	Group 2 *N* (%) = 154 (32.0)	Group 3 *N* (%) = 155 (32.2)	*p*
Age				
Child age at baseline (months), M (SD)	4.20 (1.01)	4.08 (1.04)	4.20 (1.10)	0.43
Maternal age (years), M (SD)	35.76 (7.49)	36.00 (7.15)	36.06 (7.30)	0.72
Mother's marital status				
Customary	67 (39.0)	51 (33.1)	50 (32.3)	0.99
Civil	54 (31.4)	47 (30.5)	52 (33.5)	
Religious	47 (27.7)	55 (35.7)	52 (33.5)	
Cohabiting	4 (2.3)	1 (0.6)	1 (0.6)	
Relationship to child				
Biological	172 (100)	154 (100)	155 (100)	1.00
Biological sex of the child				
Male	105 (61)	98 (63.6)	105 (67.7)	0.45
Female	67 (39)	56 (36.4)	50 (32.3)	
Diagnosis of the child				
Cerebral palsy	165 (96.0)	146 (94.8)	152 (98.1)	0.19
Spina bifida	3 (1.7)	0 (0)	0 (0)	
Hydrocephalus	1 (0.6)	2 (1.3)	1 (0.6)	
Other conditions	3 (1.7)	6 (3.9)	2 (1.3)	
Disability severity				
Mild	110 (64.0)	92 (59.7)	77 (49.7)	0.14
Moderate	38 (22.1)	35 (22.7)	45 (29.0)	
Severe	10 (5.8)	17 (11.0)	19 (12.3)	
Profound	14 (8.1)	10 (6.5)	14 (9.0)	
Family history of NDD				
Yes	31 (18.0)	29 (18.8)	27 (17.4)	0.96
No	141 (82.0)	125 (81.2)	128 (82.6)	
Residential area				
High density	104 (60.5)	97 (63.0)	107 (69.0)	0.21
Medium density	38 (22.1)	37 (24.0)	30 (19.4)	
Low density	15 (8.7)	14 (9.1)	14 (9.0)	
Peri‐urban	15 (8.7)	6 (3.9)	4 (2.6)	
Financial adequacy				
Very inadequate	4 (2.3)	8 (5.2)	10 (6.5)	0.12
Somewhat inadequate	57 (33.1)	52 (33.8)	56 (36.1)	
Moderate	93 (54.1)	86 (55.8)	82 (52.9)	
Somewhat adequate	4 (2.3)	0 (0)	0 (0)	
Very adequate	14 (8.1)	8 (5.2)	7 (4.5)	
Employment status of mother				
Formally employed	43 (25)	22 (14.3)	23 (14.8)	0.003
Self‐employed	69 (40.1)	77 (50.0)	80 (51.6)	
Unemployed	60 (34.9)	55 (35.7)	52 (33.5)	
Education level of mother				
Primary	21 (12.2)	27 (17.5)	28 (18.1)	0.62
Secondary	94 (54.7)	78 (50.6)	78 (50.3)	
Tertiary	57 (33.1)	49 (31.8)	49 (31.6)	
Medical aid cover				
Yes	31 (18.0)	32 (20.8)	30 (19.4)	0.82
No	141 (82.0)	122 (79.2)	125 (80.6)	

*Note:* Percentages are rounded and may not total 100 within groups. Values are shown as frequency (percentage), *n* (%). Family history of NDD refers to caregiver‐reported history of neuro‐developmental conditions (e.g., cerebral palsy, epilepsy or genetic developmental disorders) in first‐ or second‐degree relatives. Sociodemographic and clinical characteristics of mothers and their infants with neuro‐developmental delay across the three naturally occurring caregiver participation groups: mother‐only (Group 1), mother–father (Group 2) and mother–father–sibling (Group 3). Data are presented as mean (SD) for continuous variables and frequency (percentage) for categorical variables. Group differences were assessed using one‐way ANOVA for continuous variables and χ^2^ tests for categorical variables. Percentages are rounded and may not total 100 within groups. +Other conditions include Down syndrome, epilepsy and microcephaly.

Abbreviation: NDD = neuro‐developmental delay.

### 3.2 Developmental Outcomes Across BSID‐III Domains

Table 2 summarises the mean scores at baseline and at 3‐month follow‐up across the five BSID‐III domains. ANCOVA on follow‐up scores, adjusting for baseline, indicated significant overall group effects across all domains (all *p* < 0.05), with the strongest differences observed in the cognitive and adaptive domains. Baseline‐adjusted EMMs analyses showed that both Group 2 (Mother + Father) and Group 3 (Mother + Father + Sibling) demonstrated higher adjusted cognitive scores (*M* = 100.10 and 102.51, respectively) compared with Group 1 (Mother‐only: *M* = 98.15). Post hoc Tukey comparisons showed a significant pairwise difference for cognition between Group 3 and Group 1 (*p* < 0.05). For language, motor, socioemotional and adaptive domains, pairwise differences did not reach significance after Tukey adjustment, despite significant omnibus effects. Changes in Language, Motor and Socioemotional domains followed a similar directional trend favouring multi‐caregiver participation, but these did not reach pairwise statistical significance after multiple‐comparison adjustment. For the Adaptive domain, the omnibus ANCOVA was significant (*p* < 0.0001); however, the pairwise comparison between Groups 2 and 3 only approached significance (*p* = 0.059).

**TABLE 2:** Developmental outcomes across BSID‐III domains (baseline, three‐month and adjusted scores; *N* = 481).

Domain	Group	Baseline mean (SD)	3‐Month mean (SD)	Adjusted mean ± SE (EMM)	*p* (ANCOVA)	Interpretation
Cognitive	Mother‐only (Group 1)	97.17 (20.39)	99.67 (20.45)	98.15 ± 0.34	< 0.0001*	Significant overall group effect; Group 3 highest EMM
	Mother + Father (Group 2)	94.56 (21.71)	99.05 (22.05)	100.10 ± 0.36		
	Mother + Father + Sibling (Group 3)	95.63 (20.51)	100.18 (20.50)	102.51 ± 0.36		
Language	1	90.38 (34.80)	92.53 (34.99)	93.41 ± 0.41	< 0.0001*	Significant omnibus effect; pairwise non‐significant
	2	90.58 (33.49)	94.37 (33.85)	95.72 ± 0.40		
	3	92.79 (34.54)	98.70 (34.38)	97.84 ± 0.38		
Motor	1	97.87 (23.16)	98.69 (23.17)	98.01 ± 0.44	0.0346*	Small omnibus effect; pairwise non‐significant
	2	94.21 (24.62)	95.61 (24.86)	96.28 ± 0.42		
	3	94.79 (23.09)	96.01 (22.96)	97.05 ± 0.41		
Socioemotional	1	79.15 (29.99)	80.74 (30.38)	81.10 ± 0.37	< 0.0001*	Significant omnibus effect; pairwise non‐significant
	2	79.88 (29.02)	82.81 (29.65)	82.95 ± 0.35		
	3	78.70 (29.25)	82.58 (29.50)	83.19 ± 0.36		
Adaptive	1	108.81 (23.39)	110.05 (23.70)	109.44 ± 0.40	< 0.0001*	Omnibus significant; 2 vs. 3 approached significance (p = 0.059)
	2	104.03 (24.52)	106.95 (24.43)	111.10 ± 0.38		
	3	108.36 (23.62)	113.16 (23.50)	115.65 ± 0.37		

*Note:* Values are mean (SD) at baseline and three‐month follow‐up, with adjusted estimated marginal means (EMMs) ± standard errors from ANCOVA controlling for baseline. Group 1 = Mother‐only (reference group). Mean (SD) baseline and three‐month follow‐up scores, and baseline‐adjusted estimated marginal means (EMMs ± SE), for the five BSID‐III domains (cognitive, language, motor, socioemotional and adaptive behaviour) across the three caregiver participation groups. Group differences at follow‐up were analysed using ANCOVA adjusting for baseline scores. Omnibus *p*‐values reflect the overall group effect. Post hoc pairwise comparisons were performed using Tukey adjustment for multiple testing. Group 1 (mother‐only) is the reference category.

* *p* < 0.05 indicates statistical significance.

Regression models examined follow‐up outcomes after 3 months, adjusting for baseline scores. After adjustment, Group 3 (Mother + Father + Sibling) remained the strongest predictor of higher follow‐up cognitive scores (*β* = 4.4, 95% CI [2.2–6.6], *p* < 0.001), while Group 2 (Mother + Father) showed a smaller but still significant effect (*β* = 2.2, 95% CI [0.5–3.9], *p* = 0.01). Although several regression coefficients for the other domains showed positive trends, these effects were small in magnitude and did not consistently reach statistical significance after adjustment (Table 3). This pattern is consistent with the ANCOVA findings, where omnibus tests were significant across domains but pairwise differences were limited (Table 2). In regression models, Group 3 showed additional small but statistically significant associations with language and adaptive scores; however, these effects were not consistently supported in Tukey‐adjusted pairwise comparisons and should be interpreted cautiously.

**TABLE 3:** Multiple linear regression analysis of predictors of BSID‐III domain scores at 3‐month follow‐up (adjusted for baseline scores; *N* = 481).

Domain	Predictor group	*β* (Coefficient)*	95% confidence interval	*p*	*ηp* ^*2*^	Interpretation
Cognitive	Group 2 (Mother + Father)	2.2	0.5–3.9	0.01*	0.05	Small but significant gain versus Group 1
	Group 3 (Mother + Father + Sibling)	4.4	2.2–6.6	< 0.001*	0.09	Largest gain; approximately 2 × Group 2
Language	Group 2	2.3	−0.8–5.4	0.14	0.02	Positive, not significant
	Group 3	4.6	0.2–9.0	0.04*	0.04	Borderline‐to‐significant, approximately 2 × Group 2
Motor	Group 2	−1.7	−4.4–1.0	0.22	0.01	Small, non‐significant difference; consistent with minimal motor group effect
	Group 3	−1.0	−3.4–1.4	0.41	0.01	Small, not significant; slightly smaller than Group 2
Socioemotional	Group 2	1.5	−1.5–4.5	0.33	0.02	Small, not significant
	Group 3	3.0	−0.5–6.4	0.09	0.03	Small‐to‐moderate trend; approximately 2 × Group 2
Adaptive	Group 2	1.8	−0.6–4.2	0.15	0.03	Modest, not significant
	Group 3	6.2	1.0–11.4	0.02*	0.06	Clearer positive effect; consistent with higher EMMs

*Note:* *β* values represent baseline‐adjusted differences at 3‐month follow‐up relative to Group 1 (mother‐only). Models included the baseline score for the respective BSID‐III domain and used robust standard errors. *ηp* ^2^ values correspond to the overall group effect from the ANCOVA for that domain (i.e., 3‐group comparison), not to individual regression coefficients. Maternal employment status was not included as a covariate because baseline differences were modest, and including multiple covariates risked over‐adjustment and loss of statistical power relative to our a priori model. Results of multiple linear regression models examining the association between caregiver participation group and BSID‐III domain scores at three‐month follow‐up, adjusting for baseline domain scores.

* *p* < 0.05 indicates statistical significance.

### 3.3 Maternal Quality of Life and Mental Health

**FIGURE 1:**
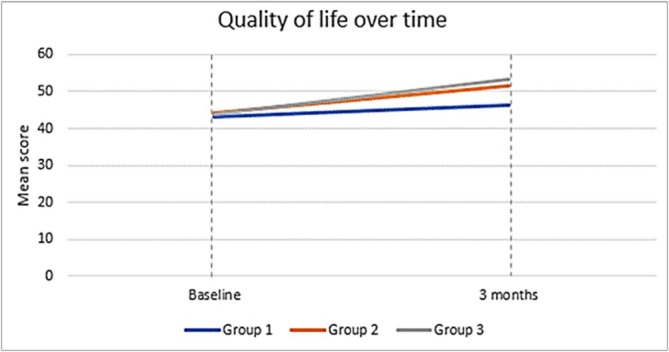
Quality of life over time on Global QoL single‐item (0–100 VAS) Trajectories of maternal Global Quality of Life (QoL) scores (0–100 visual analogue scale) at baseline and three‐month follow‐up across the three caregiver participation groups: mother‐only, mother–father and mother–father–sibling. Values represent adjusted means from repeated‐measures ANCOVA models. Improvements were greatest in the mother–father–sibling group.

**FIGURE 2:**
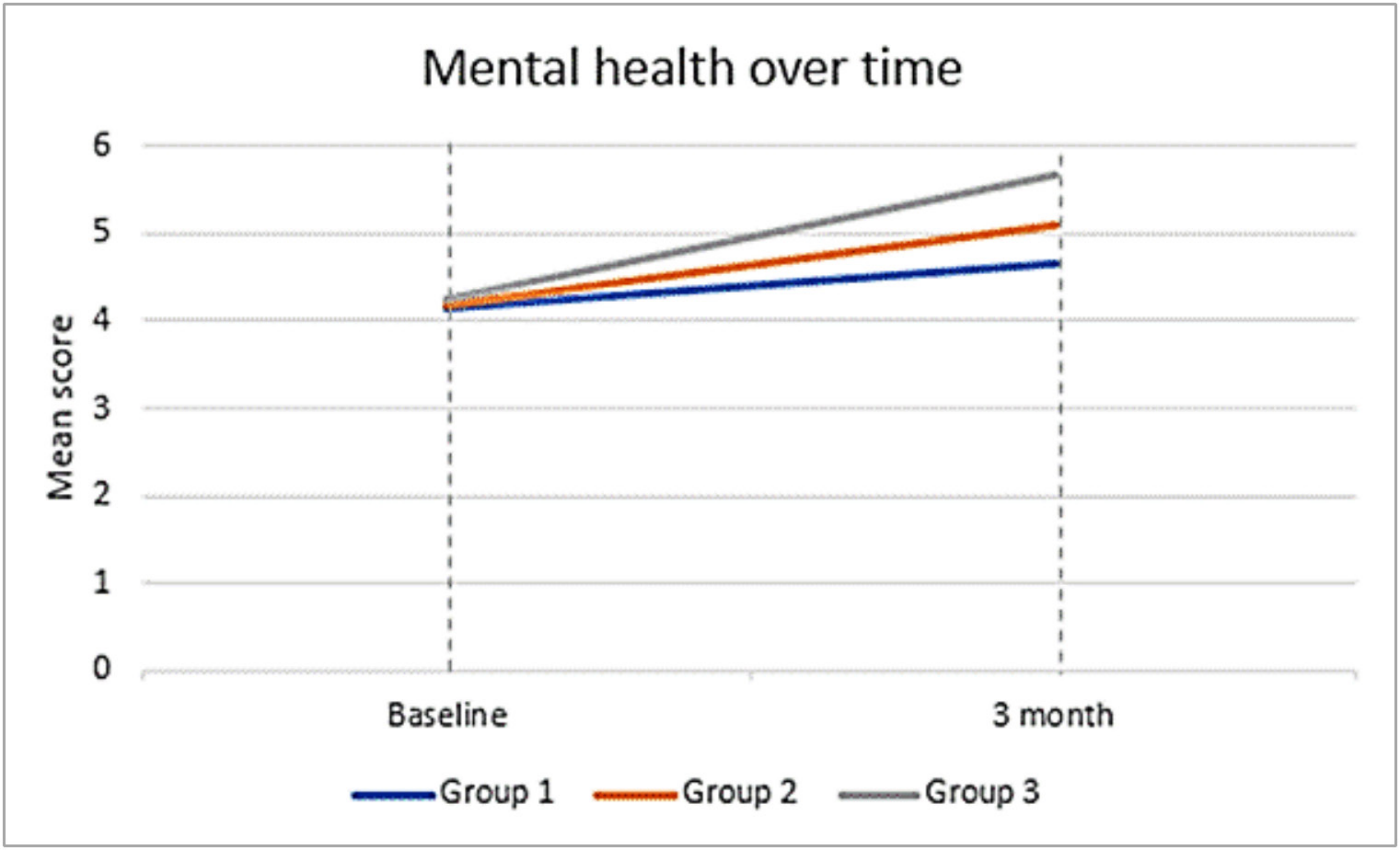
Mental health over time on Mental Health Check‐in (1–10 VAS) Trajectories of maternal mental health scores (1–10 visual analogue scale) at baseline and three‐month follow‐up across the three caregiver participation groups: mother‐only, mother–father and mother–father–sibling. Values represent adjusted means from repeated‐measures ANCOVA models. The largest improvements were observed in the mother–father–sibling group.

Mothers reported improved quality of life and mental health scores across all groups, with the largest gains observed in Group 3 (both parents and siblings involved). Repeated‐measures ANCOVA, using the time × group interaction as the primary test, revealed significant effects for both maternal mental health [*F*(2, 477) = 18.2; *p* < 0.001; *ηp* ^2^ = 0.07] and quality of life [*F*(2, 477) = 19.7; *p* < 0.001; *ηp* ^2^ = 0.08], with the greatest improvements observed in Group 3. While maternal mental health showed substantial improvement, quality of life demonstrated only small absolute gains, which nevertheless became statistically significant when the same participants were compared over time using the repeated‐measures analysis. Figures 1 and 2 depict these trajectories.

## 4 Discussion

This study examined whether extending caregiver participation beyond mothers to include fathers and siblings was associated with improved developmental outcomes and maternal well‐being, and the findings indicated selective cognitive gains in infants and meaningful improvements in maternal mental health across the three‐month period. Both the mother–father and mother–father–sibling groups demonstrated higher adjusted BSID‐III cognitive scores than the mother‐only group, with the strongest effect observed in the mother–father–sibling group. Cognitive gains were theoretically expected and methodologically supported. Maternal mental health also improved substantially, whereas quality of life scores showed only slight but significant within‐participant gains under repeated‐measures analysis. These findings extend the FCC literature linking caregiver engagement to improved child development and caregiver wellbeing (Chow et al. 2024; Jimenez‐Arberas et al. 2024). In sub‐Saharan Africa, FCC remains largely mother‐centred due to sociocultural norms and service routines that exclude other family members, resulting in reduced stimulation and caregiver strain (Nsamenang 2010; Richter and Morrell 2006). These findings suggest an implementation gap in current rehabilitation practice and provide preliminary observational evidence that multi‐caregiver participation may strengthen developmental outcomes.

The cognitive domain exhibited the clearest and most clinically meaningful group‐level change, while the language, motor, socioemotional and adaptive domains showed smaller omnibus effects that did not translate into consistent pairwise differences after Tukey adjustment, and corresponding regression effects were small, which is consistent with evidence that paternal involvement is more clearly associated with cognitive trajectories (Diniz et al. 2021; Sarkadi et al. 2008). Recent reviews emphasise that siblings may offer scaffolding and emotional companionship that enrich everyday learning opportunities (Cuskelly et al. 2023; Madzimbe et al. 2024), although unmet sibling support needs also persist even in well‐resourced settings (Veerman et al. 2025; Veerman et al. 2026). Two mechanisms explain the observed pattern. First, the dose‐and‐diversity mechanism suggests that interventions with more frequent, varied and embedded caregiver–child interactions yield stronger developmental effects (Jeong et al. 2021). Multi‐caregiver groups potentially had increased intensity and variety via father‐led challenge play, sibling‐mediated turn‐taking and maternal‐guided routines. Second, the stress–diffusion mechanism suggests that broader caregiver participation redistributes caregiving demands, reduces maternal distress, and thus enhances fidelity and consistency of implementation, aligning with the observed maternal mental health gains (Jeong et al. 2021; King et al. 2017). These mechanisms collectively illustrate how shared caregiving roles reinforce both developmental stimulation and maternal resilience.

The selective cognitive improvement, with non‐significant group‐level changes in other domains, reflects the relatively short follow‐up duration and a baseline sample skewed to milder impairment. Such a period may not allow measurable change in language or motor domains, where progress is often non‐linear and requires consolidation over extended time. Although the BSID‐III has demonstrated validity and reliability in African settings (Ballot et al. 2017; Hutchings and Potterton 2013; Rademeyer and Jacklin 2013), shorter intervals reduce measurement sensitivity, particularly in language domains. These patterns suggest that domain‐selective gains may appear first, with broader developmental changes potentially becoming evident over longer periods of sustained caregiver engagement.

Although the cognitive differences between groups were statistically significant, all three group means remained within the BSID‐III ‘average’ classification range (90–109). The approximately 4.4‐point adjusted difference between the mother‐only and mother–father–sibling groups therefore does not necessarily indicate a shift in clinical classification for most individual children. Instead, this pattern may be more meaningful at the population level, suggesting a modest dose–response relationship in which broader caregiver participation is associated with slightly higher developmental scores over a short observational period. Longer follow‐up may be required to determine whether such early differences translate into clinically meaningful developmental trajectories.

Maternal mental health improved significantly, while changes in quality of life indices were smaller than those of maternal mental health but positive and reached significance only through repeated‐measures modelling. Although no new intervention was introduced, the three groups reflected naturally occurring differences in family participation patterns that continued during the observation period. The improvements observed after 3 months likely reflect the cumulative effect of sustained father and sibling engagement. The absence of baseline differences suggests that the quality and intensity of participation, rather than its mere presence, influenced subsequent outcomes. This pattern mirrors findings from short‐term caregiver support studies, where psychological relief tends to precede observable material or functional change. The single‐item QoL VAS, though valid for overall appraisal, lacks sensitivity for detecting subtle within‐person shifts, and between‐group ANCOVA has limited power to identify such differences (Ivarsson et al. 2010). Repeated‐measures models, by contrast, can detect smaller within‐participant improvements. In our cohort, contextual factors such as high‐density living, limited medical‐aid coverage and financial strain likely constrained absolute QoL gains despite reductions in stress from shared caregiving (Lesage and Berjot 2011; Lesage et al. 2012). The findings therefore suggest a gradual but meaningful psychosocial benefit occurring within the natural course of family‐based rehabilitation.

### 4.1 Clinical Implications

Including fathers and siblings in home rehabilitation was associated with higher infant cognitive scores and improved maternal mental health, highlighting the potential importance of inclusive FCC. Although this observational study did not actively recruit or assign caregivers, the findings suggest that broader family participation in routine practice may be beneficial. Clinics could therefore consider encouraging the involvement of available caregivers during assessments and reviews to support shared responsibility in home practice. Role‐specific instructions and flexible schedules, such as after‐hours or weekend sessions, may facilitate father participation. Routine maternal mental health check‐ins with clear referral pathways may also be valuable given the observed improvements. Considering financial strain and limited medical‐aid coverage, community‐based, low‐cost interventions supported by health workers remain important for equity. However, intervention studies would be needed to confirm whether actively promoting multi‐caregiver involvement produces similar benefits. These findings may help inform discussions about incorporating father‐ and sibling‐inclusive guidance into Zimbabwe's At‐Risk Surveillance System module and other primary‐care rehabilitation guidelines.

### 4.2 Strengths and Limitations

The strengths of this study include a moderately large cohort, blinded developmental assessments and grouping based on naturally occurring family participation patterns reflective of real‐world clinical practice, which enhance external validity. Data fidelity and blinded scoring further strengthen internal validity. The replication of expected cross‐domain relationships across BSID‐III composites in LMICs adds psychometric credibility (Ballot et al. 2017; Hutchings and Potterton 2013). However, reliance on self‐reported maternal outcome measures introduces potential response bias, as perceptions of wellbeing may be influenced by social desirability or transient mood. The observational design with non‐random group allocation and potential confounding due to unmeasured variables (e.g., sibling age, temperament and home‐practice intensity) also limit causal inference. Because maternal employment status differed between groups at baseline, residual confounding by socioeconomic factors cannot be fully excluded. Variations in caregiver attendance or engagement during clinic visits could have influenced adherence to the home programme and represent a potential limitation of the observational design. Additionally, the short three‐month follow‐up may have reduced the likelihood of detecting changes in non‐cognitive domains.

### 4.3 Recommendations for Future Research

Future studies should extend follow‐up to 6–12 months to assess consolidation and sustainability of domain‐specific outcomes and incorporate fidelity or dose metrics across caregiver roles to elucidate mechanisms. Experimental or intervention studies, such as randomised designs that actively promote father and sibling participation, would also help determine whether structured multi‐caregiver involvement causally improves developmental outcomes. The use of multi‐item caregiver instruments (e.g., PedsQL Family Impact) would strengthen maternal outcome measurement. Future studies could integrate objective home‐practice logs or smartphone‐based activity trackers to quantify caregiver engagement.

## 5 Conclusion

In low‐resource urban contexts, involving fathers and siblings in home‐based rehabilitation alongside mothers was associated with selective cognitive gains and improved maternal mental health over 3 months. This longitudinal evidence supports the feasibility and potential benefits of integrating fathers and siblings into family‐centred paediatric neurorehabilitation, contributing to culturally grounded, sustainable rehabilitation models in LMICs.

## Author Contributions

**Precious Madzimbe:** conceptualization, investigation, writing – original draft, methodology, visualization, formal analysis, software, project administration, resources, writing – review and editing. **Jermaine Dambi:** writing – review and editing, methodology, supervision, validation, project administration, data curation. **Soraya Maart:** writing – review and editing, supervision, methodology, validation, project administration, data curation. **Lieselotte Corten:** writing – review and editing, supervision, methodology, validation, project administration, data curation.

## Funding

The authors have nothing to report.

## Ethics Statement

Ethical approval was obtained from the University of Cape Town Human Research Ethics Committee (HREC Approval no. HREC 482/2023) and the Medical Research Council of Zimbabwe (Approval no. MRCZ/A/3100). Written informed consent was obtained from all participating mothers for their own involvement and for the participation of their infants aged 3–6 months at baseline. Informed child assent was not age‐appropriate. Consent from fathers and siblings was not required because they were not research participants; no identifiable data were collected about them beyond caregiver‐reported participation patterns used solely for group classification. To ensure voluntariness, all participants were clearly informed that participation was entirely optional, that refusal would not affect their child's care in any way, and that clinical services would continue as usual regardless of their decision. Recruitment and data collection were conducted by trained research personnel who were not involved in the children's routine clinical management to minimise any perceived pressure. The study was non‐experimental, with no environmental manipulation, and no personal or identifiable data were collected from non‐participant family members. Confidentiality was safeguarded by anonymising data and storing files in password‐protected databases. Participants could withdraw at any point without prejudice, and referral pathways for distress or clinical concerns were in place. All procedures adhered to the ethical principles outlined in the Declaration of Helsinki and its most recent amendments (World Medical Association 2013).

## Conflicts of Interest

The authors declare no conflicts of interest.

## Data Availability

The datasets analysed during the current study are available from the corresponding author upon reasonable request.
